# FGF20 Protected Against BBB Disruption After Traumatic Brain Injury by Upregulating Junction Protein Expression and Inhibiting the Inflammatory Response

**DOI:** 10.3389/fphar.2020.590669

**Published:** 2021-01-25

**Authors:** Jun Chen, Xue Wang, Jian Hu, Jingting Du, Confidence Dordoe, Qiulin Zhou, Wenting Huang, Ruili Guo, Fanyi Han, Kaiming Guo, Shasha Ye, Li Lin, Xiaokun Li

**Affiliations:** ^1^ School of Pharmaceutical Sciences, Wenzhou Medical University, Wenzhou, China; ^2^ School of the First Clinical Medical Sciences, Wenzhou Medical University, Wenzhou, China; ^3^ Research Units of Clinical Translation of Cell Growth Factors and Diseases Research, Chinese Academy of Medical Science, Wenzhou, China

**Keywords:** Fgf20, traumatic brain injury, blood-brain barrier, tight junction protein, inflammatory response

## Abstract

Disruption of the blood-brain barrier (BBB) and the cerebral inflammatory response occurring after traumatic brain injury (TBI) facilitate further brain damage, which leads to long-term complications of TBI. Fibroblast growth factor 20 (FGF20), a neurotrophic factor, plays important roles in brain development and neuronal homeostasis. The aim of the current study was to assess the protective effects of FGF20 on TBI via BBB maintenance. In the present study, recombinant human FGF20 (rhFGF20) reduced neurofunctional deficits, brain edema, Evans blue extravasation and neuroinflammation in a TBI mouse model. In an *in vitro* TNF-α-induced human brain microvascular endothelial cell (HBMEC) model of BBB disruption, rhFGF20 reduced paracellular permeability and increased trans-endothelial electrical resistance (TEER). Both in the TBI mouse model and *in vitro*, rhFGF20 increased the expression of proteins composing in BBB-associated tight junctions (TJs) and adherens junctions (AJs), and decreased the inflammatory response, which protected the BBB integrity. Notably, rhFGF20 preserved BBB function by activating the AKT/GSK3β pathway and inhibited the inflammatory response by regulating the JNK/NFκB pathway. Thus, FGF20 is a potential candidate treatment for TBI that protects the BBB by upregulating junction protein expression and inhibiting the inflammatory response.

## Introduction

Traumatic brain injury (TBI) is a leading cause of death and disability worldwide, particularly in children and young adults (Maiti et al., 2019; Fouda et al., 2020). TBI-induced brain damage comprises two pathological phases. Primary damage occurs at the moment of trauma (Loane and Faden, 2010), while secondary damage occurs subsequently, including excitotoxicity, blood-brain barrier (BBB) disruption, oxidative stress, neuroinflammation, ischemia, edema, etc. (Polderman, 2004). Among these processes, BBB disruption partially mediated by inflammation is a vital mechanism contributing to the progression of brain injury and long-term neurological deficits (Shi et al., 2015).

The BBB plays an important role in facilitating the transmission of neuroactive agents to regulate the brain microenvironment (Wu et al., 2020), and it is disrupted by brain edema and intracranial hypertension after TBI; indeed, BBB disruption is a major cause of death (Abbott et al., 2006). The BBB is mainly composed of cerebrovascular endothelial cells (ECs) joined tightly together by intercellular tight junction (TJ) proteins and adherens junction (AJ) proteins (Chen et al., 2018; Zhu et al., 2019). The mainly barrier function is provided by podocytes projecting from astrocyte, a basement membrane, pericles, and an endothelium connected with junction proteins (Wolburg and Lippoldt, 2002). TJ and AJ proteins, including Claudin, Occludin, Zonula Occluders (ZO) and VE-cadherin, play major roles in limiting paracellular diffusion between ECs and maintaining the integrity of the BBB (Jiao et al., 2011; Zhang et al., 2020). The inflammatory reaction to TBI occurs through peripheral immune mediators produced at the entrance to the central nervous system (CNS) via a damaged BBB (Simon et al., 2017). In particular, tumor necrosis factor-α (TNF-α), interleukin (IL)-1β, IL-6 and inducible nitric oxide synthase (iNOS) are crucial proinﬂammatory cytokines produced following trauma (Aibiki et al., 1999; Kamm et al., 2006; Villalba et al., 2014) that exacerbate and increase BBB permeability (Elwood et al., 2017). Therefore, a drug targeting BBB integrity and the inflammatory response is a potential therapeutic strategy for TBI.

Fibroblast growth factor 20 (FGF20), a member of the FGF family, is a paracrine growth factor whose orthologs are highly conserved among vertebrates. Human FGF20 is highly similar to rat FGF20 and mouse FGF20 (nearly 95% amino acid identity) (Ohmachi et al., 2000; Katoh and Katoh, 2005). According to a previous study, FGF20 is predominantly expressed in the brain (Boshoff et al., 2018), has well known neurotrophic activity (Shimada et al., 2009) and plays an important role in regulating CNS development and function (Takagi et al., 2005). The positive roles of FGF20 in Parkinson’s disease (PD) imply that it may play a neuroprotective role in other CNS disorders (Niu et al., 2018). However, the effects of FGF20 on protecting BBB integrity in TBI and its possible underlying mechanism of action in regulating BBB integrity after TBI remain elusive.

Here, we aimed to determine whether recombinant human FGF20 (rhFGF20) ameliorates the pathophysiological symptoms of acute-stage TBI by preserving BBB integrity and reducing the inflammatory response. We further identified the effects of rhFGF20 on regulating the AKT/GSKβ and JNK/NFκB pathways and the roles of these pathways in preserving BBB integrity and inhibiting the inflammatory response.

## Materials and Methods

### Reagents and Antibodies

The AKT inhibitor LY294002, GSK3β inhibitor SB216763, NFκB inhibitor Bay 11–7082, FITC-dextran, and Evans blue were purchased from Sigma (Sigma-Aldrich, St. Louis, MO, United States). JNK agonist anisomycin was purchased from Beyotime (Beyotime Biotechnology, Shanghai, China). TNF-α was obtained from R&D Systems (Minneapolis, MN, United States). The following primary antibodies were applied in this study: anti-*p*-GSK3β, anti-GSK3β, anti-*p*-JNK, anti-JNK, anti-*p*-NFκB and anti-NFκB antibodies purchased from Cell Signaling Technology (Danvers, MA, United States); anti-FGF20, anti-VE-cadherin, anti-Claudin-5, and anti-GFAP antibodies obtained from Abcam (Cambridge, MA, United States); and anti-*p*-AKT, anti-AKT and anti-Occludin antibodies purchased from Invitrogen (Carlsbad, CA, United States) and Santa Cruz Biotechnology (Dallas, TX, United States), respectively. The secondary antibodies used in this study were goat anti-rabbit immunoglobulin G (IgG) H&L (HRP) and goat anti-mouse IgG-HRP purchased from Abcam (Cambridge, MA, United States) and Santa Cruz Biotechnology (Dallas, TX, United States), respectively.

### Animals and Surgical Procedures Used to Develop a Controlled Cortical Impact (CCI)-Induced TBI Model

C57BL/6 male mice (8–12 weeks, 22–28 g) purchased from the Animal Center of the Chinese Academy of Sciences (Beijing, China) were used in this study. The animal protocols were approved by the Animal Center of the Chinese Academy of Science (Shanghai, China) and the Laboratory Animal Ethics Committee of Wenzhou Medical University.

**FIGURE 1:**
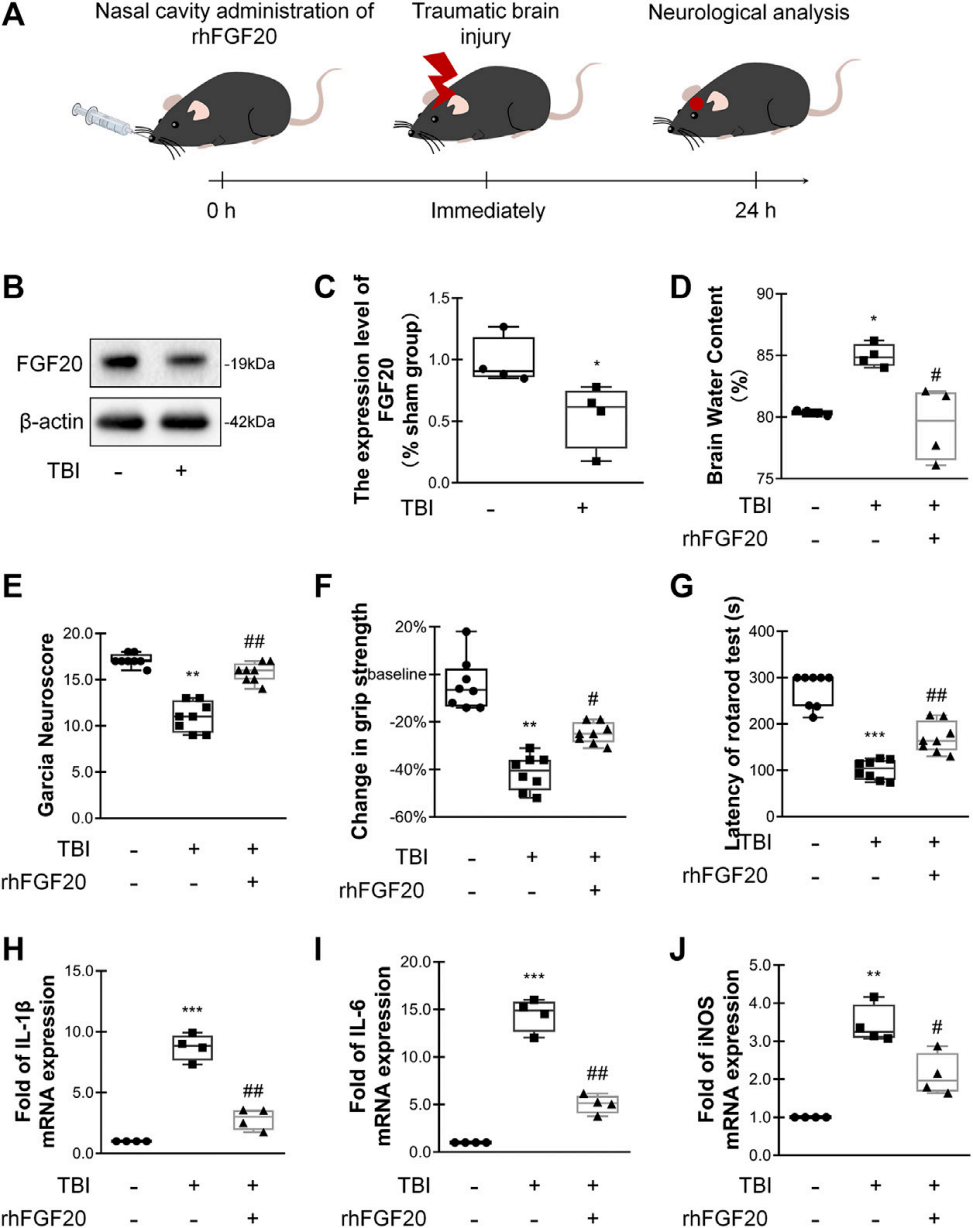
Exogenous rhFGF20 improved neurofunctional deficits and behavioral recovery in TBI mice **(A)** A schematic diagram showing the *in vivo* experimental protocol **(B)** Representative image of western blot analysis of endogenous FGF20 in the sham and TBI groups **(C)** Quantification of western blot data for endogenous FGF20 (*n* = 4) **(D)** Effects of rhFGF20 on brain water content at 24 h after TBI (*n* = 4) **(E)** Effects of rhFGF20 assessed by the Garcia test at 24 h after TBI (*n* = 8) **(F)** Effects of rhFGF20 assessed by the grip strength test 24 h after TBI (*n* = 8) **(G)** Effects of rhFGF20 assessed by the rotarod test at 24 h after TBI (*n* = 8) **(H–J)** Effects of rhFGF20 on mRNA levels of the proinflammatory cytokines IL-1β, IL-6, and iNOS, with values normalized to the housekeeping gene *β*-Actin (*n* = 4).^*^ *p* < 0.05, **p < 0.01, ***p < 0.001 vs. sham group. ^#^ *p* < 0.05, ^##^ *p* < 0.01 vs. TBI group. Data are presented as the mean values ±SD.

The surgical procedures were used to establish the controlled cortical impact (CCI)-induced TBI model with high reproducibility (Peterson et al., 2015). This model produces relevant clinical symptoms of TBI (Cernak, 2005). Male C57BL/6 mice were anesthetized with 4% isoflurane, administered normal saline or 0.5 mg/kg rhFGF20 by nasal delivery (Graff et al., 2005), and mounted on a stereotaxic frame for surgery to induce TBI. In brief, a 4-mm craniotomy was performed on the left parietal bone at the midpoint between the bregma and lambda sutures with the medial edge 0.55 mm lateral to the midline. The calvaria was carefully removed, and CCI injury was induced with a 3-mm diameter impactor at a rate of 3.0 m/s and a depth of 1.0 mm. After injury, the skin was sutured, and the temperature of the mouse was maintained at 37°C. Mice that underwent the craniotomy procedure but were not subjected to CCI were used as the sham group (Figure 1A).

### Rotarod Test

Mice were trained for 3 days before performing the Rotarod test. A ROTA ROD equipment (IITC Life Science) was set with the procedure that was gradually accelerated from 5 to 30 rpm over 5 min. This training procedure lasted for 3 days before sham and CCI surgery. The Rotarod test was performed in mice 24 h after Sham or CCI surgery, the rotarod latency was recorded as the time before the mouse fell off the rod or gripped the rod (Xu et al., 2018).

### Neurobehavioral Assessment

The Garcia test was conducted in mice 24 h after sham or CCI surgery under blinded conditions. Sensorimotor deficits were assessed using a modified Garcia test that included assessments of spontaneous activity, symmetry in the movement of the four limbs, forepaw outstretching, climbing, body proprioception and response to vibrissae touch, and was scored from 0 to 18 points, with a score of 0–three points awarded for each assessment (Garcia et al., 1995).

### Grip Strength Test

The Grip strength test was conducted in mice 24 h after surgery for Sham or CCI under blinded conditions. For grip strength measurements, each mouse was suspended over a grid by the tail such that its forepaws were allowed to grasp the grid by Grip Strength Meter (UGO BASILE biological research apparatus, Italy). The mouse was then pulled backward until it released its grip. The baseline grip strength of each animal was measured one day before CCI or the sham procedure (Glushakov et al., 2013).

### Evans Blue Extravasation

Twenty hours after sham or CCI, 0.25 ml of Evans blue dye (2% in saline) was injected intraperitoneally. Four hours after Evans blue dye injection, the anesthetized animals were transcardially perfused with saline to sufficiently remove the intravascular-localized dye and were then sacrificed. The left hemisphere was immediately weighed and homogenized in five volumes of formamide. The homogenate was incubated for 3 days at 72°C and centrifuged (15,000 rpm, 30 min). The absorbance of the supernatant was detected using a spectrophotometer (Bio-Rad, Hercules, CA, United States) at an excitation wavelength of 610 nm and an emission wavelength of 680 nm (Wang et al., 2016).

### Brain Water Content

The wet and dry weights of the brain were recorded 24 h after sham or CCI (Zhang et al., 2018). Brain tissue was obtained from mice not subjected to transcardial perfusion. The weight of the brain tissue was measured immediately after a mouse was sacrificed and was defined it as the wet weight. Subsequently, the brain was dried in an oven (100°C) for 24 h until a constant weight was achieved, and this weight was defined as the dry weight (Li et al., 2013; Genet et al., 2017). The brain water content was calculated as follow: brain water content (%) = [(wet brain weight - dry brain weight)/wet brain weight] × 100%

### Western Blot

At 24 h after CCI, mice were sacrificed and perfused with ice-cold saline solution. Brain tissue extracted from 5 mm coronal sections of the brain penumbra collected from the site of impact, and stored at −80 °C, and then were homogenized in Mammal Tissue Protein Extraction kit (Boster Biological Technology, Pleasanton, CA, United States). The cellular proteins were extracted 24 h after a TNF-α or rhFGF20 treatment by Culture Cellular Total Protein Extraction kit (Boster Biological Technology, Pleasanton, CA, United States). Supernatant was collected and protein levels were measured using the Quick Start Bradford 1X Dye Reagent (Bio- Rad, Hercules, CA, United States).

Equivalent amounts of protein were separated on 10 or 12% SDS-PAGE gels and then transferred to polyvinylidene fluoride (PVDF) membranes. Membranes were blocked with 5% nonfat milk in TBST for 90 min at room temperature and then incubated with the following primary antibodies overnight at 4°C: VE-cadherin (1:1,000), Occludin (1:1,000), Claudin-5 (1:800), *p*-AKT (1:1,000), AKT (1:1,000), *p*-GSK3β (1:1,000), GSK3β (1:1,000), *p*-NFκB (1:1,000), and NFκB (1:1,000). Membranes were subsequently incubated with the respective secondary antibodies. Finally, membranes were imaged and quantified using Image Lab 3.0 software (Bio-Rad, Hercules, CA, United States).

### Histological Analysis

Animals were anesthetized with 4% isoflurane 24 h after TBI. The whole brain was post-fixed with 4% paraformaldehyde for 12 h and embedded in paraffin. Transverse sections (5-µm thick) were mounted on slides for subsequent staining. Hematoxylin and eosin (H&E) and Nissl staining were performed to observe histopathological changes according to the manufacturer’s protocol (Vigo et al., 2019). Images were acquired using a light microscope.

Immunofluorescence staining was performed to detect protein expression. For *in vivo* protein detection, sections were deparaffinized, rehydrated, and incubated with 5% bovine serum albumin (BSA) to block nonspecific binding. Then, sections were incubated with primary antibodies overnight at 4°C. The following primary antibodies were applied: VE-cadherin (1:300), Occludin (1:300), Claudin-5 (1:300), Iba-1 (1:100), and glial fibrillary acidic protein GFAP (1:300). For the negative control slides, primary antibody was replaced with 1% BSA. The sections were incubated with Alexa Fluor 488-conjugated secondary antibodies (1:500) for 1 h at room temperature. Then the nuclei were stained with DAPI. The stained sections were imaged using a Nikon ECLPSE 80i fluorescence microscope (Nikon, Japan). No immunoreactive cell was observed in the negative control slides.

ImageJ (NIH, United States) was used to automatically determine the fluorescence intensity, Soma area and process length of microglia (Iba-1^+^ cells) and astrocytes (GFAP^+^ cells) from microscopic fields randomly selected from the peri-injury regions of four animals per group.

### RNA Extraction and RT-PCR

Total RNA was extracted from 5-mm brain coronal sections prepared from the site of impact or HBMECs using the TriPure Isolation Reagent (Roche, South San Francisco, CA, United States). The RNA concentration was quantified by NanoDrop spectrophotometer (Thermo Fisher Scientific, MA, United States). One microgram of RNA was used to synthesize cDNAs with the PrimeScript RT Reagent Kit (RR037A, TaKaRa, Japan), and then PCR was conducted with the SYBR Green PCR Master Mix (Bio- Rad, Hercules, CA, United States). The oligonucleotide PCR primer pairs are listed in Table 1, purchased from Sango Biotech (Shanghai, China). The relative quantity in each sample was normalized to the level of β-Actin mRNA expressed. The results are presented as the means ± SEMs of duplicate samples from three independent experiments.

**TABLE 1:** Primers’ sequences used for real-time PCR analysis.

Gene	Sequence 5′-3′	Amplication length (bp)
IL-1β (mouse)	AAG​CCT​CGT​GCT​GTC​GGA​CC	140
	TGA​GGC​CCA​AGG​CCA​CAG​GT	
TNF-α (mouse)	CAA​GGG​ACA​AGG​CTG​CCC​CG	109
	GCA​GGG​GCT​CTT​GAC​GGC​AG	
IL-6 (mouse)	AGA​AGG​AGT​GGC​TAA​GGA​CCA​A	101
	AAC​GCA​CTA​GGT​TTG​CCG​AGT​A	
β-Actin (mouse)	CAC​TGC​AAA​CGG​GGA​AAT​GG	198
	TGA​GAT​GGA​CTG​TCG​GAT​GG	
IL-1β (human)	GCC​CTA​AAC​AGA​TGA​AGT​GCT​CCT	104
	CCT​GAA​GCC​CTT​GCT​GTA​GTG	
TNF-α (human)	GTG​ACA​AGC​CTG​TAG​CCC​A	414
	ACT​CGG​CAA​AGT​CGA​GAT​AG	
IL-6 (mouse)	ACT​CAC​CTC​TTC​AGA​ACG​AAT​TG	149
	CCA​TCT​TTG​GAA​GGT​TCA​GGT​TG	
β-Actin (human)	AGC​ACA​ATG​AAG​ATC​AAG​AT	188
	TGT​AAC​GCA​ACT​AAG​TCA​TA	

### Cell Culture and *in vitro* BBB Disruption Model

Primary human brain microvascular endothelial cells (HBMECs; ACBRI376, Cell Systems Corporation, Kirkland, WA) were cultured in endothelial cell basal medium-2 (EBM-2) and plated in 6-mm wells or 24-well Transwell inserts coated with collagen I. The plates were incubated at 37°C in a humidified atmosphere containing 5% CO_2_. Recombinant human TNF-α was used to establish the BBB disruption model (Rochfort and Cummins, 2015). Cells at a confluence of approximately 90% were treated with TNF-α (50 ng/ml), rhFGF20 (50 nM) or in combination with the AKT inhibitor LY294002 (20 μM) (Wang et al., 2016), the GSK3β inhibitor SB216763 (30 μM) (Li et al., 2015), the JNK agonist anisomycin (4 μM) (Li et al., 2012), or the NFκB inhibitor Bay11–7082 (5 μM) (Odobasic et al., 2019) for 24 h and were then harvested for further analysis.

### FITC-Dextran Permeability

HBMECs were cultured at a density of 1 × 10^5^ cells/well on polycarbonate 24-well Transwell insert chambers with 0.4-mm pores. After 2 days, cells had proliferated and reached compact density, and then were treated with or without TNF-α and rhFGF20. After 24 h, the medium from the Transwell inserts was replace with new medium containing 0.1 mg/ml of fluorescein isothiocyanate (FITC)-labeled dextran (MW, 70,000; Sigma, St. Louis, MO). After 4 h, the fluorescence of each well was detected with spectrophotometer (Bio-Rad, Hercules, CA, United States) at an excitation wavelength of 485 nm and an emission wavelength of 520 nm (Zhao et al., 2019).

### Trans-endothelial Electrical Resistance (TEER)

TEER indicating the permeability of the HBMEC monolayer was measured with an EVOM resistance meter (World Precision Instruments, Sarasota, FL) according to the operation manual. Twenty-four-well Transwell inserts (6.5 mm, 0.4-mm pores) with cultured HBMECs were transferred to a chamber containing 0.1 M KCl based on a previous study (Lin et al., 2018). Then, the culture medium in the Transwell insert was replaced with 0.1 M KCl. The chamber and Transwell insert were connected through an EndOhm connector cable. The resistance was then measured with the EVOM resistance meter, and the TEER value was multiplied by the total membrane surface area to obtain the resistance value in Ω⋅cm^2^. A blank Transwell chamber without HBMECs was used as the blank control (Zhao et al., 2019).

### Statistical Analysis

GraphPad Prism software (GraphPad Software, Inc., San Diego, CA) was used to analyze the data. Statistically significant differences among data from three or more groups were evaluated using one-way analysis of variance (ANOVA) followed by the Tukey-Kramer test. *p* < 0.05 was considered statistically significant.

## Result

### rhFGF20 Treatment Improved Neurofunctional and Behavioral Recovery in Mice After TBI

In order to assess the relationship between FGF20 expression and acute-stage TBI, we investigated the levels of FGF20 protein in the mouse brain. Compared with the sham group, the FGF20 level in the brain of the TBI group was significantly reduced (Figure 1B,C). These data suggested that FGF20 has a certain correlation with TBI.

We assessed the degree of brain edema following TBI by analyzing the brain water content. The brain water content was significantly decreased in mice in the rhFGF20 treatment group compared to mice in the TBI group 24 h after TBI (Figure 1D). After TBI, the neurological function of the mice was severely damaged. We then investigated whether rhFGF20 promoted behavioral recovery following TBI. The Garcia test was used to assess neurofunctional deficits in mice with TBI (Garcia et al., 1995). Mice exhibited neurofunctional deficits after TBI, and rhFGF20 administration markedly ameliorated these neurofunctional deficits (Figure 1E). The grip strength test was used to assess forelimb function (Glushakov et al., 2013). Mice in the TBI group exhibited functional deficits in forelimb strength; however, administration of rhFGF20 diminished these functional deficits (Figure 1F). The rotarod test measures motor coordination in mice. Latency in the rotarod test was decreased in mice in the TBI group, whereas rhFGF20 clearly increased the latency (Figure 1G). These results indicated that exogenous rhFGF20 ameliorated neurofunctional deficits, increased forelimb strength, reduced motor coordination behavioral deficits and decreased the degree of cerebral edema after TBI.

### rhFGF20 Inhibited the Inflammatory Response After TBI

TBI induces an inflammatory response that increases the levels of several inflammatory factors, producing further damage. To evaluate the anti-inflammatory effect of rhFGF20 after TBI, we examined the levels of the proinflammatory cytokines IL-1β, IL-6 and the proinflammatory enzyme iNOS after TBI.

The expression levels IL-1β, IL-6, and iNOS were measured using RT-PCR. The administration of rhFGF20 significantly reduced the expression of these proinflammatory cytokines after TBI (Figure 1H–J). Based on the data presented above, we postulated that the rhFGF20 treatment inhibited inflammation and protected against BBB disruption after TBI.

### rhFGF20 Treatment Improved Brain Tissue Survival in Mice Following TBI

**FIGURE 2:**
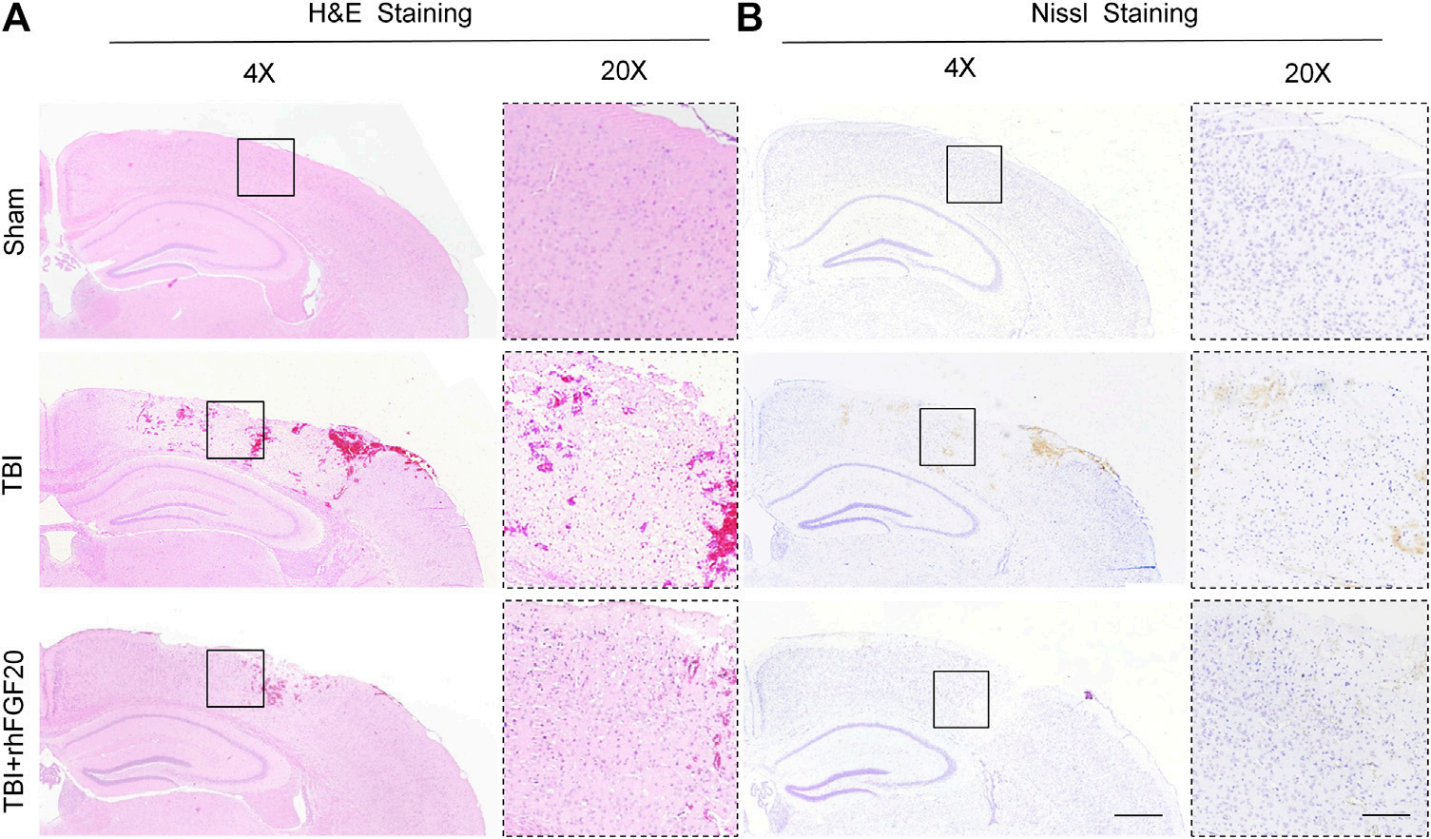
Exogenous rhFGF20 enhances brain tissue survival in TBI mice **(A)** Effects of rhFGF20 on damaged brain tissue after TBI, as visualized by H&E staining **(B)** Effects of rhFGF20 on neurons in the brain after TBI, as demonstrated by Nissl staining. 4×: Bar, 500 μm. 20×: Bar, 100 μm (*n* = 4).

Furthermore, Hematoxylin-eosin (H&E) staining and Nissl staining of brain sections were performed to assess the effect of rhFGF20 on the brain tissue and neurons. H&E staining revealed the cells in the brain tissue of the sham group mice were arranged neatly and the cytoplasm of the cells was abundant. However, many pyknotic cells were observed in the brain tissue of TBI-induced mice. In addition, the cytoplasm presented light coloring. While rhFGF20 administration partially ameliorated these abnormalities (Figure 2A). In addition, Nissl staining showed that rhFGF20 decreased neuronal loss after TBI (Figure 2B).

### rhFGF20 ameliorated TBI-Induced BBB Disruption and Increased the Expression of Junction Proteins

**FIGURE 3:**
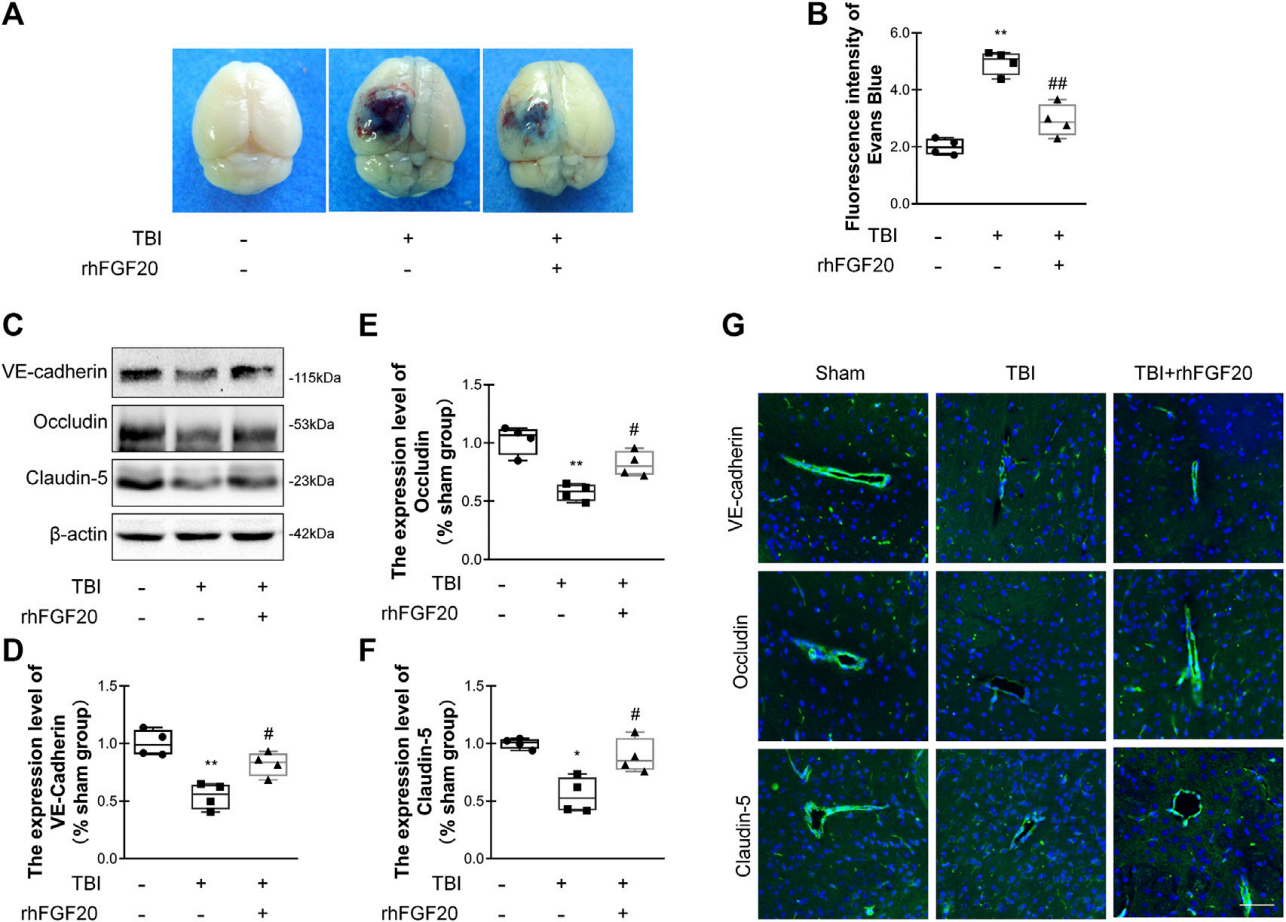
Exogenous rhFGF20 protected against BBB disruption in the mouse brain 24 h after TBI **(A)** Representative images of Evans blue extravasation **(B)** Effects of rhFGF20 on Evans blue fluorescence (*n* = 4) **(C)** Representative image of western blot analysis of VE-cadherin, Occludin, and Claudin-5. *β*-Actin was used as the loading control and for band density normalization **(D–F)** Quantification of western blotting data for VE-cadherin, Occludin, and Claudin-5 protein (*n* = 4) **(G)** Effects of rhFGF20 on the expression of the VE-cadherin, Occludin, and Claudin-5 analyzed by immunofluorescence. The nuclei are labeled with DAPI (*n* = 4). Bar, 50 μm. Magnification, ×40. *p < 0.05, **p < 0.01 vs. sham group. ^#^ *p* < 0.05, ^##^ *p* < 0.01 vs. TBI group. Data are presented as the mean values ±SD.

Evans blue dye can leak though the damaged BBB and remain in the cortex, and Evans blue extravasation experiments can elucidate the degree of BBB disruption (Chen et al., 2017). Compared with the sham group, Evans blue dye extravasation was significantly increased in the TBI group, while treatment with rhFGF20 dramatically reduced leakage (Figure 3A,B), indicating that rhFGF20 ameliorated TBI-induced BBB damage.

TJ and AJ proteins are important mediators of BBB integrity. To elucidate the effect of rhFGF20 on TJ and AJ proteins, we determined junction protein levels after TBI via western blot analysis and immunofluorescence staining. Western blot analysis revealed significantly decreased levels of VE-cadherin, Occludin and Claudin-5 after TBI. In contrast, rhFGF20 markedly increased the levels of these proteins (Figure 3C–F). The results of immunofluorescence staining were consistent with the western blotting data (Figure 3G). These data indicated that rhFGF20 treatment protected against BBB disruption after TBI and increased the expression of TJ and AJ proteins.

### rhFGF20 Inhibited Neuroinflammation and Suppressed Glial Activation After TBI

**FIGURE 4:**
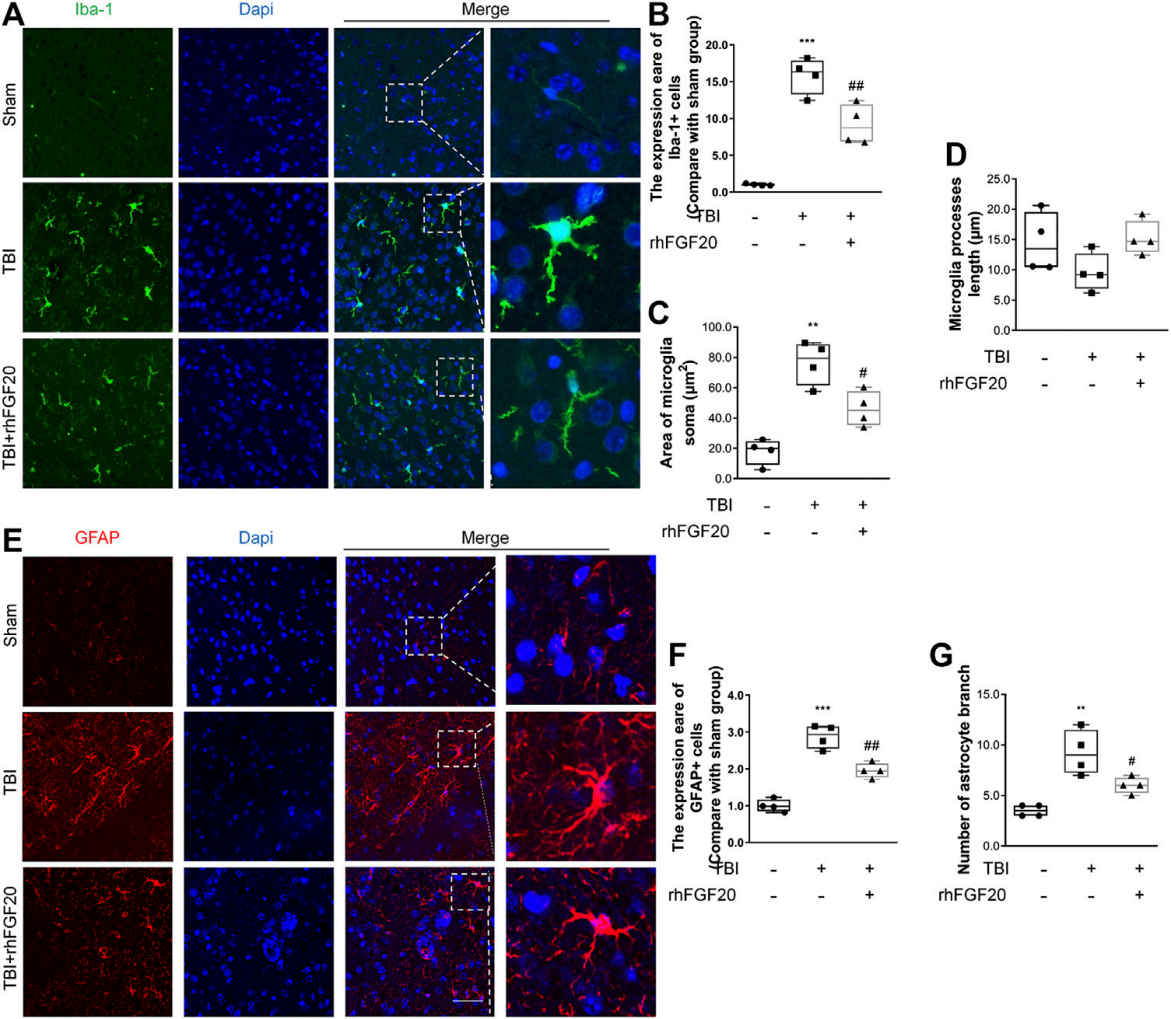
Exogenous rhFGF20 inhibited neuroinflammation in the mouse brain 24 h after TBI **(A)** Representative images of microglial activation. Nuclei are labeled with DAPI **(B–D)** Quantification of immunofluorescence staining data showing the expression of Iba-1-positive microglial, microglial Soma area and microglial process length (*n* = 4) **(E)** Representative images of astrocytes activation. Nuclei are labeled with DAPI **(F–G)** Quantification of immunofluorescence staining data showing the expression of GFAP-positive astrocytes, and astrocytes branches number (*n* = 4). Bar, 50 μm **p < 0.01, ***p < 0.001 vs. sham group. ^#^ *p* < 0.05, ^###^ *p* < 0.001 vs. TBI group. Data are presented as the mean values ±SD.

Activation of microglial and astrocytes activation, a key manifestation of CNS neuroinflammation (Schober et al., 2019), was assessed using immunofluorescence labeling to determine the cell number, soma area, and process length. TBI clearly activated Iba1-positive microglia in the cortex, as indicated by the increased soma area compared with those in the sham group. However, rhFGF20 treatment inhibited neuroinflammation by reducing the number of activated microglia and soma area, however, microglial process length was not significantly affected (Figure 4A–D). In addition, TBI greatly increased the number of activated GFAP-positive cells in the cortex and the number of branches, while rhFGF20 treatment significantly decreased the population of activated astrocytes and the number of their branches (Figure 4E–G).

### rhFGF20 Improved Recovery After TBI via the AKT/GSK3β and JNK/NFκB Signaling Pathways

**FIGURE 5:**
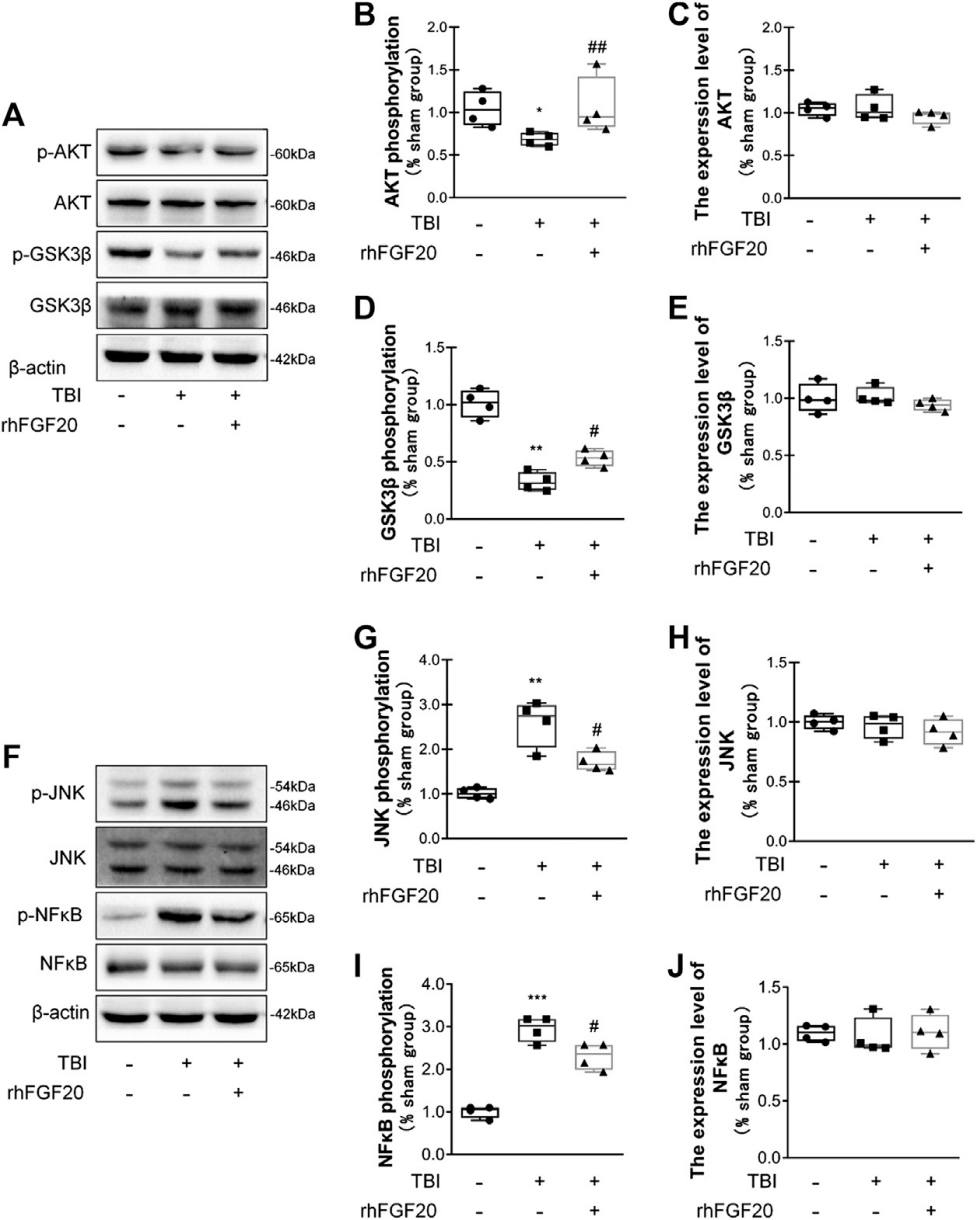
Exogenous rhFGF20 improved recovery after TBI via the AKT/GSK3β and JNK/NFκB signaling pathways **(A)** Representative image of western blot analysis of *p*-AKT, AKT, *p*-GSK3β and GSK3β in the mouse brain 24 h after TBI **(B–E)** Effects of rhFGF20 on *p*-AKT, AKT, GSK3β and *p*-GSK3β protein levels, as analyzed by quantification of western blot data (*n* = 4) **(F)** Representative images from western blot analysis of *p*-JNK, JNK, *p*-NFκB and NFκB. *β*-Actin was used as the loading control and for band density normalization **(G–J)** Effects of rhFGF20 on the *p*-JNK, JNK, *p*-NFκB and NFκB protein levels, as analyzed by quantification of western blotting data (*n* = 4).*p < 0.05, **p < 0.01, ***p < 0.001 vs. sham group. ^#^ *p* < 0.05, ^##^ *p* < 0.01 vs. the TBI group. Data are presented as the mean values ±SD.

In the current study, to investigate the mechanism underlying the protective effect of rhFGF20 after TBI, we evaluated downstream pathway proteins. According to a previous study, the TBI-induced decrease in junction protein levels correlates with the AKT pathway (Wang et al., 2016). Our data showed that rhFGF20 administration significantly reversed the TBI-induced decrease in the phosphorylation of AKT and GSK3β, however, the expression level of total AKT and total GSK3β didn't obviously changed. (Figure 5A–E). These data suggested that AKT/GSK3β are probably involved in the protective effect of rhFGF20 on the BBB.

JNK plays an important role in inflammatory diseases (Zhou et al., 2019) and the transcription factor NFκB also is a major regulator of inflammation. Activation of JNK was determined by monitoring its phosphorylation. In addition, NFκB pathway activation was assessed by detecting the level of its phosphorylation. We detected the activation levels of JNK and NFκB in our study. In mice subjected to TBI, the levels of JNK phosphorylation and NFκB phosphorylation were both increased. However, rhFGF20 decreased the levels of *p*-JNK and *p*-NFκB after TBI, while both TBI surgery and FGF20 administration did not significantly affect the total expression level of JNK and NFκB (Figure 5F–J). These data suggest that JNK/NFκB is probable involved in the anti-inflammatory effect of rhFGF20.

### rhFGF20 Protected Against TNF-α-Induced BBB Injury

The most important component and functional unit of BBB is endothelial cells. The TNF-a-induced BBB injury model on HBMEC was specifically used in this study for the protective effect of rhFGF20 in ECs, and then verified that our previous conjecture that FGF20 can repair the BBB injury and its specific mechanism.

**FIGURE 6:**
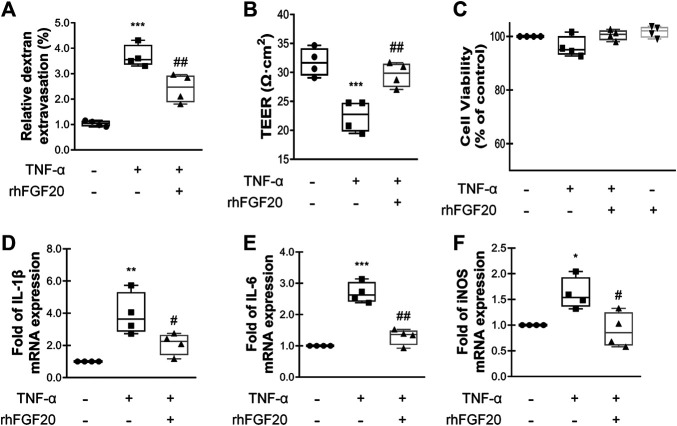
rhFGF20 protected against TNF-α-induced BBB injury **(A)**Effects of rhFGF20 in the HBMECs FITC-dextran permeability test (*n* = 4) **(B)** Effects of rhFGF20 on TEER measurements (*n* = 4) **(C)** Effects of TNF-α and rhFGF20 on HBMEC viability (*n* = 4) **(D–F)** Effects of rhFGF20 on the mRNA levels of the proinflammatory cytokines IL-1β, IL-6, and proinflammatory enzyme iNOS; levels were normalized to those of the housekeeping gene *β*-actin (*n* = 4). **p < 0.01, ***p < 0.001 vs. the control group. ^#^ *p* < 0.05, ^##^ *p* < 0.01 vs. the TNF-α group. Data are presented as the mean values ±SD.

To show the paracellular permeability of ECs, FITC-dextran permeation through the monolayer was assessed, and TEER experiments were performed. The TNF-α group showed a high degree of FITC-dextran permeation degree and low resistance, indicating that the barrier integrity was severely damaged. However, rhFGF20 treatment clearly reduced FITC-dextran permeation and ameliorated the decrease in resistance (Figure 6A,B). In addition, the results of the Cell Counting Kit-8 (CCK8) cell viability assay suggested that TNF-α and rhFGF20 did not regulate the cell survival rate (Figure 6C). Therefore, the effect of rhFGF20 on the TNF-α-induced disruption of endothelial monolayer permeability was mediated by treating damaged cells rather than increasing the number of cells. We postulate that the TNF-α-induced cellular model of BBB disruption used in our study was a reasonable approach and that rhFGF20 treatment ameliorated TNF-α-induced endothelial barrier damage. The expression levels of IL-1β, IL-6 and iNOS were significantly increased in TNF-α-induced HBMECs; however, rhFGF20 exerted an anti-inflammatory effect on TNF-α-induced HBMECs by decreasing the levels of these inflammatory factors (Figure 6D-F).

### rhFGF20 Protected Against TNF-α-Induced BBB Injury by Increasing Junction Protein Expression

**FIGURE 7:**
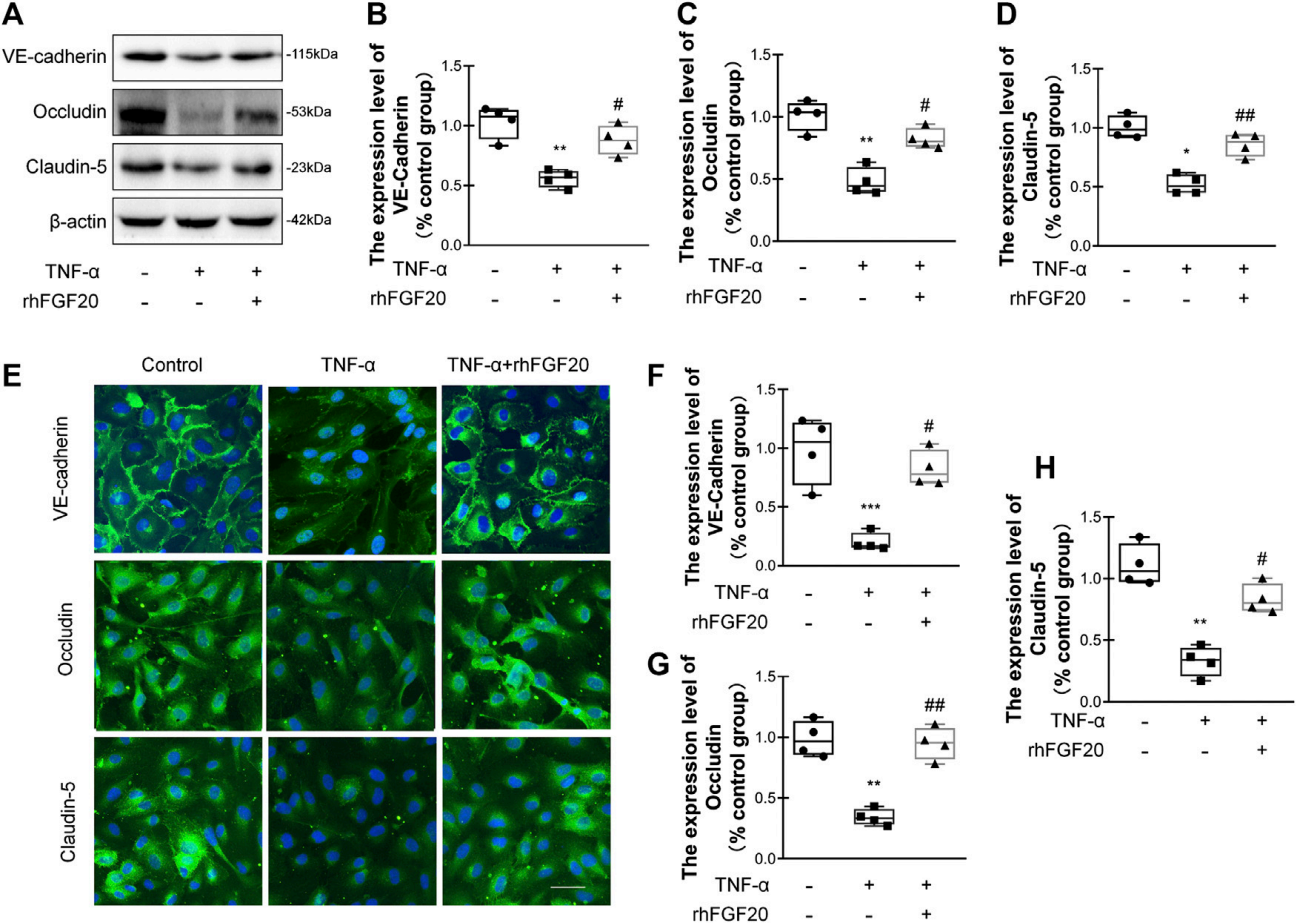
rhFGF20 protected against TNF-α-induced BBB injury by increasing junction protein expression **(A)** Effects of rhFGF20 on the expression of VE-cadherin, Occludin, and Claudin-5, as assessed by western blot analysis of total cellular protein. *β*-Actin was used as the loading control and for band density normalization **(B–D)** Effects of rhFGF20 on VE-cadherin, Occludin, and Claudin-5 protein levels, as analyzed by quantification of western blot data (*n* = 4) **(E)** Effects of rhFGF20 on the expression of VE-cadherin, Occludin, and Claudin-5. Nuclei are labeled with DAPI **(F–H)** Effects of rhFGF20 on VE-cadherin, Occludin, and Claudin-5 protein levels, as analyzed by quantification of immunofluorescence staining data (*n* = 4). Bar, 50 μm. Magnification, ×40. *p < 0.05, **p < 0.01, ***p < 0.001 vs. the control group. ^#^ *p* < 0.05, ^##^ *p* < 0.01 vs. the TNF-α group. Data are presented as the mean values ±SD.

TJ and AJ proteins are important regulators of paracellular permeability in ECs. Therefore, we detected the levels of TJ and AJ proteins in HBMECs. TNF-α significantly decreased the levels of VE-cadherin, Occludin and Claudin-5; however, rhFGF20 increased the levels of these proteins (Figure 7A–D). The results of immunofluorescence staining were consistent with the western blotting. TNF-α significantly decreased the levels of these junction proteins in the membrane, whereas rhFGF20 administration dramatically rescued the levels of these proteins (Figure 7E–H).

### rhFGF20 Protected Against TNF-α-Induced BBB Injury by Activating the AKT/GSK3β Signaling Pathway

**FIGURE 8:**
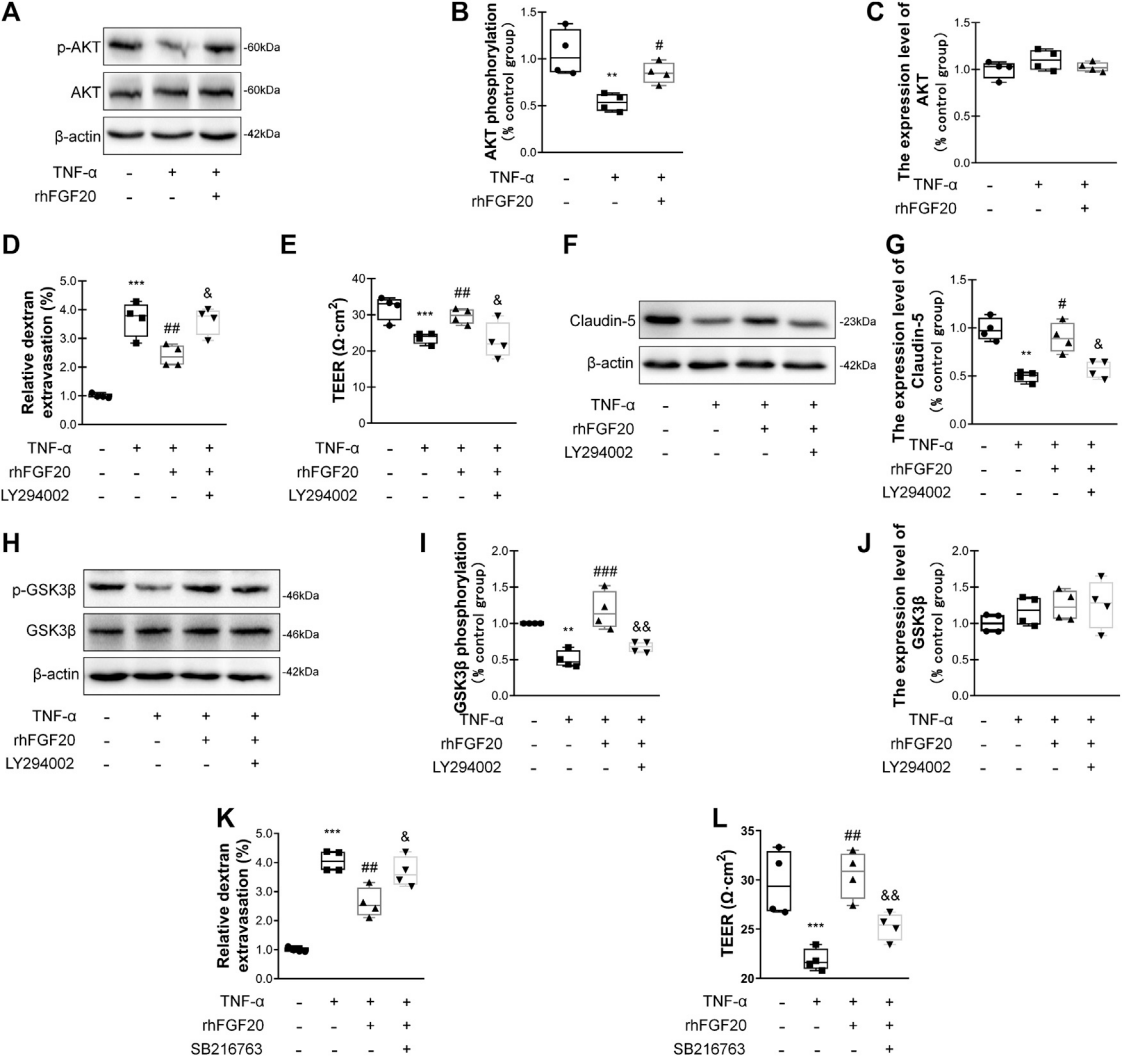
rhFGF20 ameliorated HBMEC monolayer disruption by increasing junction protein expression via AKT/GSK3β activation **(A)** Effects of rhFGF20 on *p*-AKT and AKT levels, as shown by western blot analysis of cellular protein, with *β*-actin used as the loading control and for band density normalization **(B–C)** Effects of rhFGF20 on *p*-AKT and AKT protein levels, as analyzed by quantification of western blot data (*n* = 4) **(D–E)** The AKT inhibitor LY294002 (20 μM) reversed the rhFGF20-mediated rescue of changes in FITC-dextran transport and decreased endothelial resistance (*n* = 4) **(F–G)** LY294002 blocked the effect of rhFGF20 on Claudin-5 expression, as shown by western blot analysis of total cellular protein, with *β*-actin used as the loading control and for band density normalization (*n* = 4) **(H–J)** Effects of rhFGF20 and LY294002 on *p*-GSK3β and GSK3β levels, as shown by western blot analysis of total cellular protein, with *β*-actin used as the loading control and for band density normalization (*n* = 4) **(K–L)** The GSK3β inhibitor SB216763 (30 μM) reversed the rhFGF20-mediated rescue of changes in FITC-dextran transport and decreased endothelial resistance (*n* = 4). **p < 0.01, ***p < 0.001 vs. the control group. ^#^ *p* < 0.05, ^##^ *p* < 0.01, ^###^ *p* < 0.001 vs. the TNF-α group. ^&^ *p* < 0.05, ^&&^ *p* < 0.01 vs. TNF-α + rhFGF20 group. Data are presented as the mean values ±SD.

We evaluated whether the effects of rhFGF20 on TNF-α-induced BBB injury were mediated by the AKT/GSK3β pathway. Consistent with the *in vivo* results, rhFGF20 significantly rescued the TNF-α-induced decrease in AKT phosphorylation. And the expression level of total AKT didn't observably changed after TNF-α treatment or rhFGF20 treatment (Figure 8A–C). The AKT inhibitor LY294002 was administered with rhFGF20 to determine whether the protective effect of rhFGF20 on the BBB observed in our study is related to AKT. LY294002 not only significantly reversed the effect of rhFGF20 on restoring BBB permeability and endothelial resistance (Figure 8D,E) but also decreased the expression of Claudin-5 (Figure 8F,G). We further assessed the effect of rhFGF20 downstream of AKT and found that LY294002 significantly inhibited the rhFGF20-induced increase in the level of *p*-GSK3β but not total GSK3β (Figure 8H–J). The GSK3β inhibitor SB216763 also reversed the protective effect of rhFGF20 on permeability and endothelial resistance (Figure 8K–L). These results further confirmed that rhFGF20 protected against TNF-α-induced BBB injury by activating the AKT/GSK3β signaling pathway.

### rhFGF20 Protected Against TNF-α-Induced BBB Injury by Inhibiting the Inflammatory Response Mediated by the JNK/NFκB Signaling Pathway

**FIGURE 9:**
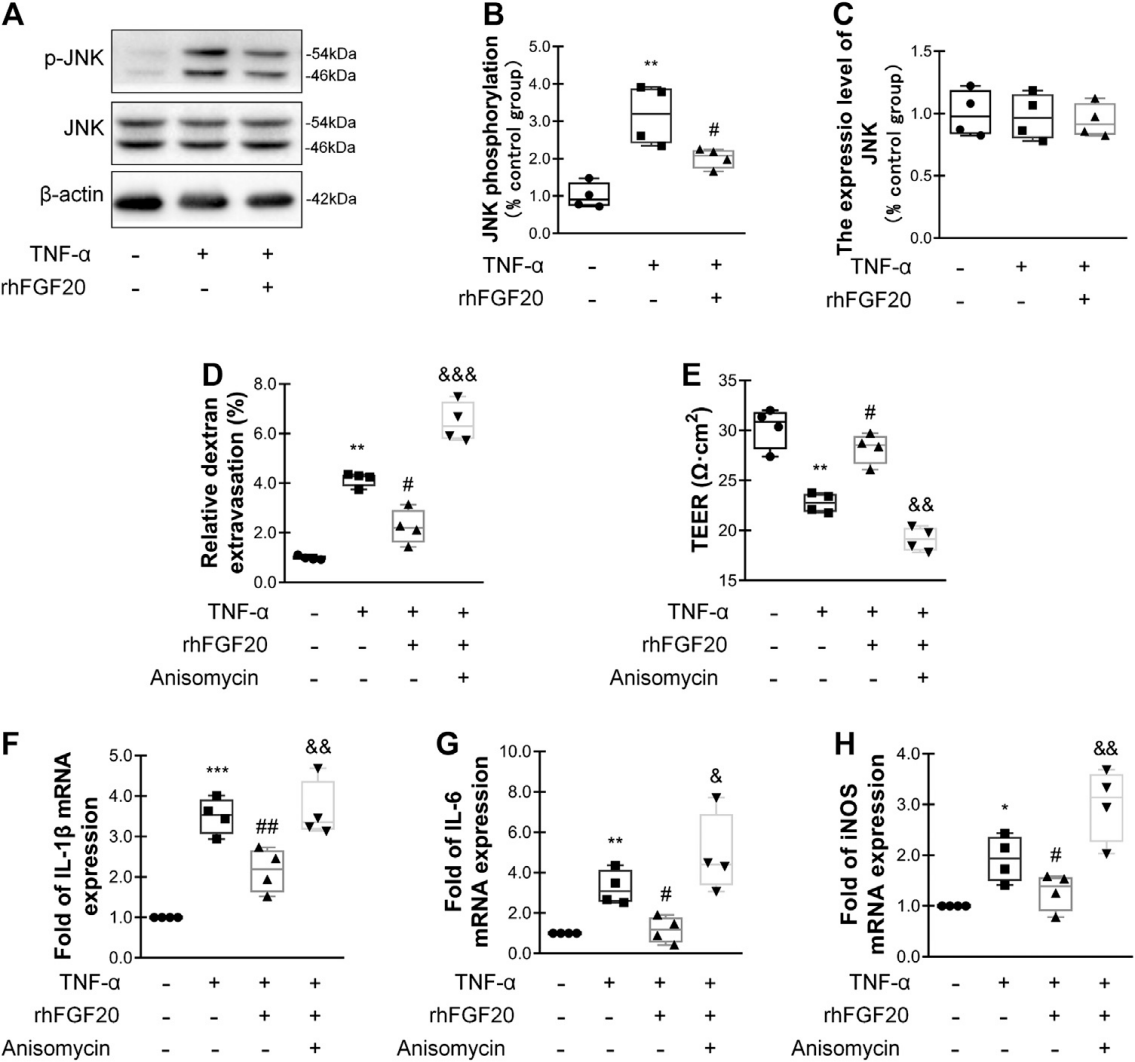
rhFGF20 ameliorated HBMEC monolayer disruption by inhibiting the inflammatory response via the JNK/NFκB signaling pathway **(A)** Effects of rhFGF20 on *p*-JNK and JNK levels, as shown by western blot analysis, with *β*-actin used as the loading control and for band density normalization **(B–C)** Effects of rhFGF20 on *p*-JNK and JNK protein levels, as analyzed by quantification of western blot data (*n* = 4) **(D–E)** The JNK agonist anisomycin (20 μM) reversed the rhFGF20-mediated rescue of changes in FITC-dextran transport and decreased endothelial resistance (*n* = 4) **(F–H)** Anisomycin blocked the effect of rhFGF20 on the mRNA levels of the proinflammatory cytokines IL-1β, IL-6, and iNOS (*n* = 4). *p < 0.05, **p < 0.01, ***p < 0.001 vs. the control group. ^#^ *p* < 0.05, ^##^ *p* < 0.01 vs. the TNF-α group. ^&^ *p* < 0.05, ^&&^ *p* < 0.01, ^&&&^ *p* < 0.001 vs. the TNF-α + rhFGF20 group. Data are presented as the mean values ±SD.

**FIGURE 10:**
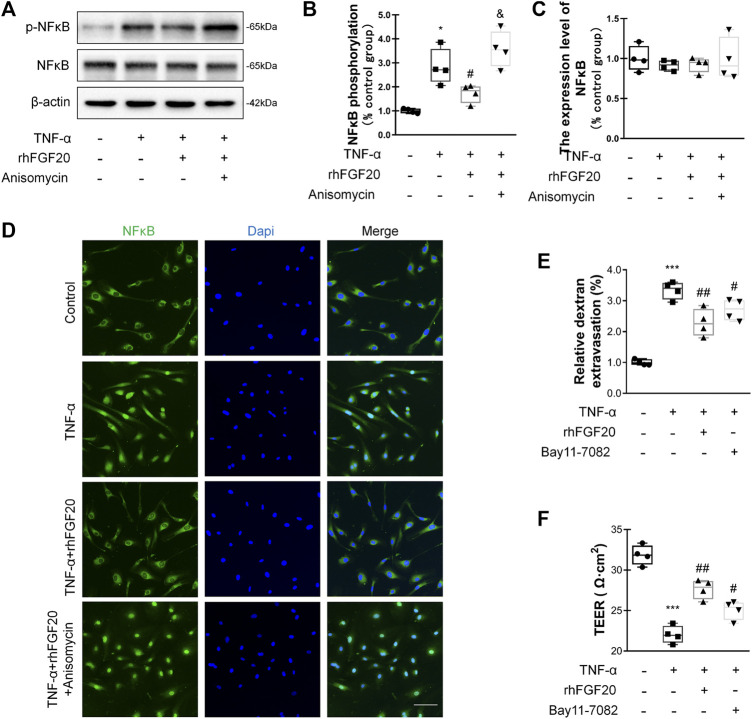
rhFGF20 ameliorated HBMEC monolayer disruption by decreasing the inflammatory response via the JNK/NFκB signaling pathway **(A)** Effects of rhFGF20 and anisomycin on *p*-NFκB and NFκB levels, as shown by western blot analysis of the total cellular protein, with *β*-actin used as the loading control and for band density normalization **(B–C)** Effects of rhFGF20 and anisomycin on *p*-NFκB and NFκB protein levels, as analyzed by quantification of western blot data (*n* = 4) **(D)** Effects of rhFGF20 and anisomycin on NFκB activity. Nuclei are labeled with DAPI (*n* = 4) **(E–F)** The NFκB inhibitor Bay 11–7082 also reversed TNF-α-induced increase in BBB permeability and decrease in endothelial resistance (*n* = 4). *p < 0.05, ***p < 0.001 vs. the control group. ^#^ *p* < 0.05, ^##^ *p* < 0.01 vs. the TNF-α group. ^&^ *p* < 0.05, vs. TNF-α + rhFGF20 group. Data are presented as the mean values ±SD.

In addition, we investigated the anti-inflammatory mechanism by which rhFGF20 protects against BBB injury. The JNK signaling pathway is important for endothelial inflammation (Lu et al., 2019). Consistent with the *in vivo* results, rhFGF20 significantly decreased the TNF-α-induced increase in JNK phosphorylation, but did not affect the expression level of total JNK (Figure 9A–C). Furthermore, we used the JNK agonist anisomycin in our study. Anisomycin not only significantly reversed the effect of rhFGF20 on restoring the BBB permeability and endothelial resistance (Figure 9D,E) but also increased the level of the IL-1β, IL-6 and iNOS mRNAs (Figure 9F–H). The downstream targets in the JNK signaling pathway include various transcription factors. The transcription factor NFκB is a major regulator of inflammation. We speculated that NFκB is a downstream factor in the JNK pathway. We further assessed the anti-inflammatory effects of rhFGF20 downstream of JNK and found that anisomycin significantly inhibited the rhFGF20-induced decrease in NFκB phosphorylation and nuclear translocation, but did not significantly affect the total NFκB expression, as detected using both western blotting and immunofluorescence staining (Figure 10A–D). The NFκB inhibitor Bay 11–7082 was applied to examine the effect of NFκB on endothelial monolayer permeability. Bay 11–7082 also reversed the TNF-α-induced injury to BBB permeability and endothelial resistance (Figure 10E,F). These results further confirmed that rhFGF20 protected against TNF-α-induced BBB injury by decreasing the inflammatory response mediated by the JNK/NFκB signaling pathway.

## Discussion

**FIGURE 11:**
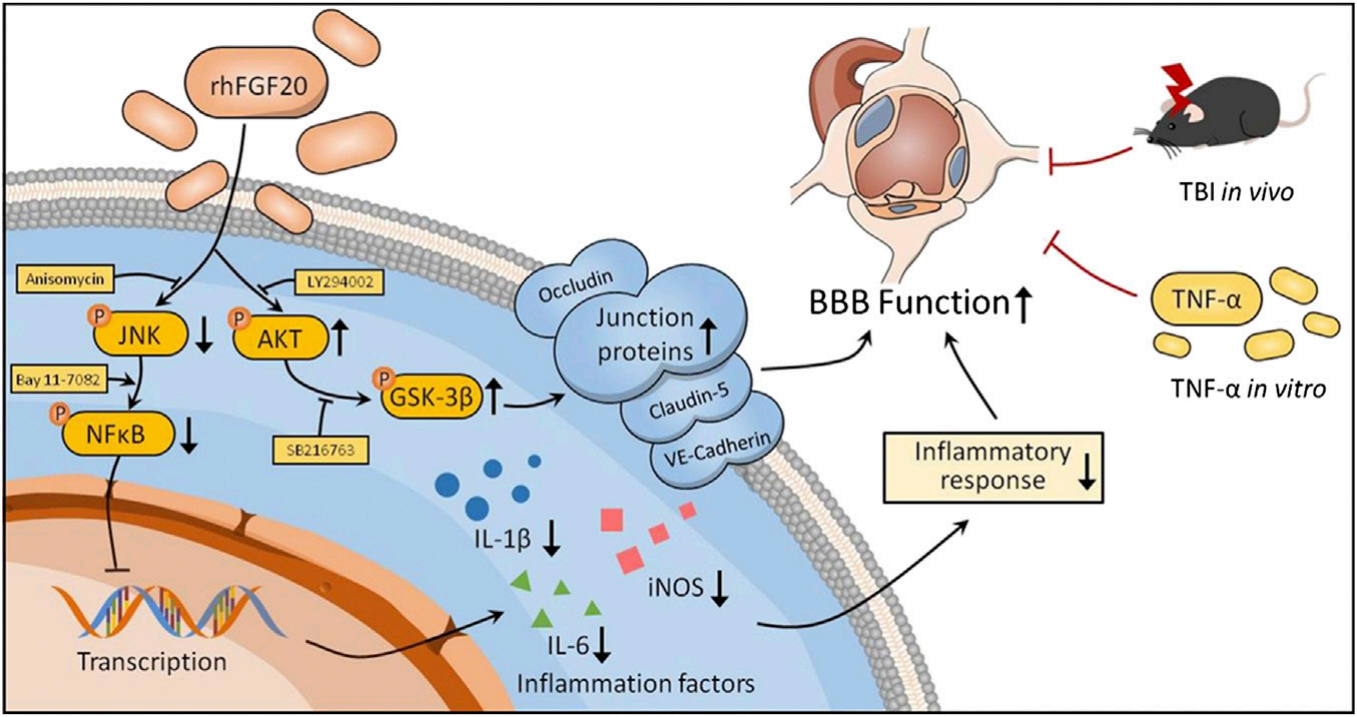
Potential signaling pathways involved in rhFGF20-mediated protection against BBB disruption both *in vivo* in TBI mice and an *in vitro* in a TNF-α-induced HBMEC model. rhFGF20 upregulates junction proteins via the AKT/GSK3β pathway and inhibits proinflammatory cytokine expression by suppressing the JNK/NFκB pathway, eventually protecting against BBB disruption.

BBB dysfunction occurs within hours or days after TBI and contributes to secondary injury processes, such as edema, cell death, and neuroinflammation, as well as to the severity of the injury (Main et al., 2018). BBB breakdown can be a therapeutic target in TBI treatment (Shlosberg et al., 2010). In the current study, we confirmed that exogenous administration of rhFGF20 upregulated the expression of TJ and AJ proteins VE-cadherin, Occludin and Claudin-5, protected against BBB disruption, decreased the degree of cerebral edema, thereby ameliorating functional behavior deficits after TBI. In addition, *in vitro* TNF-α-induced HBMECs were established to model BBB disruption. Similarly, rhFGF20 upregulated the expression of the TJ and AJ proteins, and ameliorated the TNF-α-induced increase in HBMEC permeability. Both the *in vivo* and *in vitro* studies demonstrated that rhFGF20 upregulated the expression of TJ and AJ proteins and protected against BBB disruption by activating the AKT/GSK3β pathway. In addition, exogenous administration of rhFGF20 inhibited inflammation in ECs through the JNK/NFκB signaling pathway. These data demonstrated that rhFGF20 might be useful as a therapeutic agent for BBB protection and TBI treatment (Figure 11).

Emerging studies have demonstrated that FGFs have positive effects after TBI. Overexpression of FGF2 promoted neurogenesis and protected neurons against degeneration in the hippocampus of adult mice after TBI (Yoshimura et al., 2003). Exogenous administration of FGF2 has been reported to attenuate histopathological damage and improve functional outcomes after TBI (Yan et al., 2000). Mesenchymal stem cells (MSCs) overexpressing FGF21 (MSC-FGF21) transplanted to mouse brain by intracerebroventricular injection have been shown to significantly alleviated TBI-induced spatial memory deficits, and enhanced hippocampal neurogenesis (Shahror et al., 2020). In our previous study, rhFGF21 administration markedly upregulated TJ and AJ protein expression, preserved BBB integrity, and reduced neurofunctional behavior deficits and cerebral edema in a mouse model after TBI (Chen et al., 2018). The present study is the first to reveal the capacity of FGF20 to preserve the BBB after TBI and to identify the underlying mechanism of action.

The BBB is composed of microvascular ECs and TJ proteins (Occludin, Claudins, and ZO) and adhesion junction proteins (VE-cadherin and PECAM-1) that interconnect with ECs (Citi and Cordenonsi, 1998; Stamatovic et al., 2008). TJ protein expression in brain ECs at a relatively high level, can maintain the integrity of the BBB (Zhang et al., 2020), which is crucial for brain homeostasis. In contrast, disruption of BBB integrity correlates with reduced expression of TJ proteins such as Occludin, Claudin-5, and ZO (Nag et al., 2007; Williams et al., 2020). Mechanical stress associated with acute-stage TBI not only is detrimental to cells in the nervous system but also results in injury to the microvasculature in the brain, which consists of ECs, finally increasing paracellular permeability and causing a cascade of secondary damage to brain tissue (Alvarez et al., 2011). Therefore, we analyzed the protective effects of rhFGF20 on VE-cadherin, Occludin, and Claudin-5 expression in the brains of TBI mice and *in vitro* in TNF-α induced HBMECs by western blotting and immunofluorescence assays.

FGF20, expressed mainly in the brain, has been suggested to be a critical factor involved in brain development and neuronal homeostasis (Dono, 2003). Reports about the therapeutic effects of FGF20 on CNS conditions, except for PD and Alzheimer's disease (AD), are limited. FGF20 exerted a protective effect on dopamine neurons in a rat model of 6-hydroxydopamine-induced PD (Boshoff et al., 2018; Fletcher et al., 2019). However, the role of FGF20 in the BBB after TBI is unknown. In the present study, endogenous FGF20 levels were significantly decreased after TBI; this altered expression of endogenous FGF20 might be associated with TBI and could be beneficial in regulating the repair process. Therefore, the post-TBI protective effects of rhFGF20 on the BBB were explored. Considering that FGF20 cannot penetrate the BBB, it was administered by nasal delivery based on a previous study (Wang et al., 2016) before CCI-induced TBI; this method is a major limitation of this study. In subsequent studies, we will perform further experiments to adjust the time of the treatment window for FGF20 administration after TBI, which will make the findings more clinically meaningful.

In addition to being affected by primary damage resulting from the initial impact, alterations in BBB integrity can also be influenced by deleterious secondary injury responses, including inflammatory cascades and abnormal metabolic processes within the CNS (Maiti et al., 2019). These inflammatory factors supplement TJ destruction in the BBB (Ye et al., 2013). Several studies have reported that BBB dysfunction leads to a high degree of cerebral edema, increases the mortality rate and changes behavior (Xu et al., 2017; Chen et al., 2018).

Neuroinflammation involving the activation of microglia and astrocytes has been demonstrated to play a critical role in the secondary injury induced by TBI (Schober et al., 2019). In addition, treatment with FGF20 significantly inhibited the expression of Iba1 and GFAP markers of microglia and astrocyte activation, respectively 24 h after TBI. FGF functions through FGF receptor (FGFR) activation (Kalinina et al., 2009; Romero-Fernandez et al., 2011). Moreover, a previous study reported that FGFR1 also is also expressed in astrocytes (Narvaez et al., 2020) and microglia (Tang et al., 2018) in the brain. A recent study reported that FGF21 suppressed microglia/macrophage-mediated neuroinflammation in stroke (Wang et al., 2020a). Herein, we speculated that FGF20 might have the potential to regulate glial cells in order to control neuroinflammation in brain disease. However, the effect of FGF20 on glial cells and the underlying mechanism will be explored in our next work after we gain a better understanding of the protective effect of FGF20 after TBI. The AKT pathway is necessary for cell survival, and an increase in the *p*-AKT level protects against BBB damage after TBI (Wang et al., 2016). Phosphorylation of AKT decreases GSK3 activity (Zhao et al., 2006) by increasing the phosphorylation of GSK3β. Phosphorylation of GSK3β results in increased expression of neuroprotective and neurotrophic proteins (Ren et al., 2003) and brain-derived neurotrophic factor (BDNF) (Yasuda et al., 2009) and attenuates BBB recovery (Yu et al., 2012). Reports indicate that other FGFs can activate FGFR1 in ECs, regulate the AKT pathway, and exert cytoprotective effects (Huang et al., 2012). In the present study, we concluded that rhFGF20 increased the expression of junction proteins by activating the AKT/GSK3β pathway, and then preserved the function of the BBB. However, the precise mechanism by which FGF20 regulates the expression of junction proteins through the AKT/GSK3β pathway remains elusive. We speculate that several potential mechanisms, including increasing protein synthesis, regulating protein modification and reducing protein degradation, may be involved, and we will explore and verify these mechanisms in the future.

Although more evidence is focused on BBB disruption after TBI, various factors affect BBB permeability, such as the animal species, the injury models, the severity of impact, and permeability measurements (Main et al., 2018). However, the extent and time of BBB permeability after TBI remain poorly understood. Main et al. (2018) thoroughly documented the extent and time of BBB permeability after TBI in different species and with detection methods. Acute BBB dysfunction appeared at 12–76 h with different measurement methods, and in some cases up to months or years post injury in humans. Rats exhibited BBB permeability at acute time points of 30 min-24 h, extending to 3 days after TBI in only a few individuals. Similarly, mouse models showed that acute BBB disruption began from 6 h and extended to 3 days; many pharmacological effects were evaluated at a single time point, most often 24 h post injury. In the present study, we established a CCI mouse model and explored the effects of rhFGF20 on TBI after 24 h of treatment. Our results showed that exogenous administration of rhFGF20 attenuated BBB disruption and the neuroinflammatory response in mice after TBI. In addition, rhFGF20 administration alleviated cerebral edema and ameliorated functional behavior deficits after TBI.

Many mechanisms, including autophagy (Kim et al., 2020), endoplasmic reticulum stress (Xu et al., 2019), oxidative stress (Sharma et al., 2020) and so on, are involved in BBB damage. However, all of these mechanisms are related to inflammation, indicating that direct inhibition of inflammation also plays an important role in the repair of the BBB. We also found changes in cortical tissue post TBI and in TNF-induced HBMECs. In the TBI mouse model, rhFGF20 reduced the levels of inflammatory cytokines including IL-1β, IL-6, and iNOS. In addition, we further conducted *in vitro* model experiments and confirmed that FGF20 has a direct anti-inflammatory effect on TNF-α-induced ECs. The above results indicated that FGF20 can ameliorate ECs damage by inhibiting EC inflammation and then repair the BBB. In addition, we found that rhFGF20 markedly inhibited microglial activation in cortical tissue post TBI, as indicated by the reduced numbers of activated cells and somal areas, and increased ramified processes.

NFκB is a crucial transcription factor involved in the generation of the inflammatory response (Wang et al., 2020b). In addition, JNK is important for NFκB transactivation or translocation, which plays a pivotal role in inﬂammation process and constitutes an optimal therapeutic target for the pathogenesis of inﬂammation (Lan et al., 2019). We further explored whether the JNK signaling pathway was involved in the anti-inflammatory mechanism of rhFGF20. In the present study, administration of rhFGF20 to TBI mice and TNF-α-treated HBMECs decreased the level of *p*-JNK, and inhibited NF-κB activity and neuroinflammatory factor expression. Moreover, the JNK agonist anisomycin inhibited the anti-inflammatory effect of rhFGF20 and its ability to evoke repair of EC permeability. In addition, the NFκB inhibitor Bay11–7082 showed protective effects on EC permeability. Collectively, the above results demonstrated that rhFGF20 protected against BBB disruption by suppressing the inflammatory response, an effect that might be mediated through inhibition of the JNK and NF-κB signaling pathways. aFGF has been reported to inhibit the activity of JNK and NFκB through TAK/TAB to exert anti-inflammatory effects, and FGF21 can inhibit the JNK/NFκB pathway (Lee et al., 2012); however this group did not mention activation of FGFR1. Therefore, we believe that FGF20 plays a protective role in ECs, and regulates the AKT pathway by activating FGFR1. In addition, FGF20 may also play a role in inhibiting the JNK signaling pathway through other receptors, a possibility that needs further study.

## Conclusion

In conclusion, these experimental findings demonstrated that treatment with exogenous rhFGF20 protected against BBB disruption by maintaining the integrity of TJ and AJ protein expression by activating the AKT/GSK3β pathway. In addition, rhFGF20 alleviated the cerebral inflammatory response by regulating the JNK/NFκB pathway, facilitating neurofunctional recovery and decreasing the degree of brain edema after TBI. These results were confirmed *in vitro* in TNF-α induced HBMECs (Figure 11). These findings extended our understanding of the effects of FGF20 in TBI treatment and suggested that FGF20 has potential clinical applications in cerebral endothelial barrier disruption-related disease and can be a therapeutic candidate for TBI.

## Data Availability Statement

Publicly available datasets were analyzed in this study. This data can be found here: The raw data supporting the conclusions of this article will be made available by the authors, without undue reservation, to any qualified researcher.

## Ethics Statement

The animal study was reviewed and approved by Laboratory Animal Centre, Wenzhou Medical University.

## Author Contributions

LL and XL designed the experiments. JC and XW were involved in the design of the described experiments. JC, JH, WH, CD, KG, FH, and RG carried out the experiments. SY and JD analyzed data, QZ compiled the figures. JC and SY wrote the manuscript. XW and LL reviewed the manuscript. All authors read and approved the final manuscript.

## Funding

This work was supported by the by the National Natural Science Foundation of China (No. 81971180), Medical and Health Science and Technology Program of Zhejiang Province (2018KY505, WKJ-ZJ-2130), Natural Science Foundation of Zhejiang Province (LQ19H090012), CAMS Innovation Fund for Medical Sciences (2019-I2M-5-028), and Wenzhou Municipal Science and Technology Bureau (Y20170686).

## Conflict of Interest

The authors declare that the research was conducted in the absence of any commercial or financial relationships that could be construed as a potential conflict of interest.
